# Deletion of the transcriptional regulator TFAP4 accelerates c-MYC-driven lymphomagenesis

**DOI:** 10.1038/s41418-023-01145-w

**Published:** 2023-03-09

**Authors:** Margaret A. Potts, Shinsuke Mizutani, Alexandra L. Garnham, Connie S. N. Li Wai Suen, Andrew J. Kueh, Lin Tai, Martin Pal, Andreas Strasser, Marco J. Herold

**Affiliations:** 1 The Walter and Eliza Hall Institute of Medical Research, Blood Cells and Blood Cancer Division, Parkville, VIC Australia; 2 Department of Medical Biology, University of Melbourne, Parkville, VIC Australia; 3 Present Address: Division of Hematology and Oncology, Department of Medicine, Kyoto Prefectural University of Medicine, Kyoto, Japan; 4 Present Address: School of Dentistry and Medical Sciences, Charles Sturt University, Wagga Wagga, NSW Australia

**Keywords:** Cancer, Immunology

## Abstract

Many lymphoid malignancies arise from deregulated c-MYC expression in cooperation with additional genetic lesions. While many of these cooperative genetic lesions have been discovered and their functions characterised, DNA sequence data of primary patient samples suggest that many more do exist. However, the nature of their contributions to c-MYC driven lymphomagenesis have not yet been investigated. We identified TFAP4 as a potent suppressor of c-MYC driven lymphoma development in a previous genome-wide CRISPR knockout screen in primary cells in vivo [1]. CRISPR deletion of TFAP4 in *Eµ-MYC* transgenic haematopoietic stem and progenitor cells (HSPCs) and transplantation of these manipulated HSPCs into lethally irradiated animals significantly accelerated c-MYC-driven lymphoma development. Interestingly, TFAP4 deficient *Eµ-MYC* lymphomas all arose at the pre-B cell stage of B cell development. This observation prompted us to characterise the transcriptional profile of pre-B cells from pre-leukaemic mice transplanted with *Eµ-MYC/Cas9* HSPCs that had been transduced with sgRNAs targeting TFAP4. This analysis revealed that TFAP4 deletion reduced expression of several master regulators of B cell differentiation, such as *Spi1*, *SpiB* and *Pax5*, which are direct target genes of both TFAP4 and MYC. We therefore conclude that loss of TFAP4 leads to a block in differentiation during early B cell development, thereby accelerating c-MYC-driven lymphoma development.

## Introduction

The oncogene *c-MYC* is abnormally highly expressed *in* ~70% of human cancers [2]. Notably, c-MYC is commonly overexpressed in several haematological malignancies, such as Burkitt lymphoma (BL), a B cell malignancy driven by the t(8;14) chromosomal translocation that subjugates the *c-MYC* gene under the control of the immunoglobulin heavy (*IgH*) chain gene enhancer *Eµ* [3]. The *Eµ-MYC* transgenic mouse serves as a pre-clinical model of BL and other malignancies driven by deregulated expression of c-MYC. These mice constitutively express human *c-MYC* under the control of the murine *Eµ* enhancer throughout B cell development leading to increased expansion of B cell progenitors that subsequently transform into lymphoma following the acquisition of cooperating oncogenic mutations [4–6].

Since the development of BL and other lymphoid malignancies requires additional genetic or epigenetic aberrations that cooperate with deregulated *c-MYC* expression, we previously set out to identify such cooperating mutations in an unbiased genome-wide functional genetic screen in vivo [1]. From this screen, we identified the transcription factor TFAP4 (also known as AP-4) as one of the top hits [1]. TFAP4 is a basic helix-loop-helix leucine zipper transcription factor reported to both activate or repress target gene expression upon homo-dimerisation [7]. *Tfap4* expression itself is directly regulated by c-MYC. TFAP4 contributes to several c-MYC regulated cellular processes, including cell proliferation, cell cycle control and cellular senescence, by functional interaction with c-MYC including binding to the same gene promoter elements (reviewed by Wong et al. [8]). Of note, TFAP4 is critical for sustaining c-MYC-driven proliferation and maturation signals in B cells of the germinal centres following an infectious challenge through binding and regulating the same gene promoters [9]. Thus, TFAP4 is an important transcription factor acting downstream of and/or in parallel with c-MYC to regulate the proliferation and Ig class switching of mature B lymphoid cells.

TFAP4 has predominantly been identified as an oncogene that has been implicated in driving tumourigenesis by impacting the epithelial to mesenchymal transition, “stemness” and other cellular processes [8]. In certain human cancers high levels of TFAP4 expression correlate with poor prognostic outcomes [10, 11]. However, these oncogenic properties of TFAP4 were found in solid cancers, such as breast cancer [12], colorectal carcinoma [13, 14], gastric cancer [15], neuroblastoma [16, 17] and certain others [18, 19], and the role of TFAP4 in haematological malignancies has only been investigated in one study to date [20]. Interestingly, mutations in *TFAP4* were identified in 9.7% of BL patient samples [21–23]. Conversely to solid cancers (see above), these were predominantly inactivating mutations, suggesting TFAP4 has tumour suppressive functions in this context. However, this type of analysis does not provide mechanistic insights into how defects in TFAP4 contribute to c-MYC-driven lymphomagenesis.

We employed *Eµ-MYC*/*Cas9* doubly transgenic mice and a fetal liver derived HSPC transplantation approach to investigate the tumour suppressive role of TFAP4 during c-MYC-driven lymphoma development. We found that loss of TFAP4 led to malignant transformation at the pre-B cell stage, prior to surface Ig (sIg) expression. Gene expression analysis of pre-leukaemic pre-B cells revealed that several transcription factors important for B cell differentiation that, are also direct targets of TFAP4 and MYC, were abnormally reduced when TFAP4 was absent. This demonstrates that TFAP4 binds to and regulates key genes responsible for B cell differentiation and maturation. In *Eµ-MYC* transgenic B lymphoid cells the loss of TFAP4 dysregulates differentiation resulting in expansion of the rapidly proliferating pre-B cell pool that transforms into fully malignant c-MYC driven lymphoma.

## Materials and methods

### Cell culture and lentivirus production

Recipes for the culture media for HEK293T cells and *Eµ-MYC* lymphoma cell lines (FMA medium) derived from primary tumours are listed in Supplementary Table S1 [24]. Cell lines were routinely tested for mycoplasma contamination. Some *sgControl/Eµ-MYC/Cas9* lymphoma cell lines were previously used for experiments in [1]. Lentiviruses were generated using standard calcium phosphate precipitation methods as previously described [25]. Supernatants containing lentivirus particles were collected 48–72 h after transfection and passed through a 0.45 µM filter prior to infection of cells.

### Experimental mice

The care and husbandry of experimental mice was conducted according to the guidelines outlined by The Walter and Eliza Hall Institute of Animal Ethics Committee. *Eµ-MYC* transgenic [4] and *Cas9* transgenic mice (a gift from Prof K. Rajewsky, Max Delbrueck Centre, Berlin, Germany) were maintained on a C57BL/6-Ly5.2 background. C57BL/6-Ly5.1 and C57BL/6-Ly5.2 mice were obtained from the Walter and Eliza Hall Institute breeding facility (Kew, Victoria, Australia).

### Haematopoietic reconstitution of lethally irradiated mice

Fetal liver cells (FLCs), a rich source of HSPCs, from *Eµ-M*YC/*Cas9* double transgenic E13.5 embryos were harvested and frozen in 90% FCS and 10% (v/v) DMSO. Prior to transduction with sgRNA expression vectors, FLCs were thawed and cultured for 48 h in FLC medium (Supplementary Table S1). Twelve-well non-tissue culture treated plates were coated with 32 µg/ml retronectin (WEHI) in PBS overnight at 4 °C and then blocked with 2% bovine serum albumin solution (#A1595 Sigma-Aldrich) in PBS at 37 °C for 30 min. Viral supernatant supplemented with 8 µg/ml polybrene was centrifuged onto the retronectin coated plates at 3500 rpm for 2 h at 32 °C. Supernatant was removed and replaced with FLCs to be transduced for 24 h. These transduced cells were then collected, washed in PBS and injected intra-venously (i.v.) (random assignment of donor cells) into lethally irradiated (2 × 5.5 Gy, 4 h apart) 7–8 week-old C57BL/6-Ly5.1 recipient mice. Tumour-free survival was defined as the time from HSPC transplantation until the animal developed lymphoma and was deemed to have reached ethical endpoint by experienced animal research technicians who were blinded to the identity of the donor HSPCs used for transplantation of this mouse.

### Analysis of pre-leukaemic pre-B cells

Reconstitution of lethally irradiated mice with FLCs was performed as described above, except that cells from one fetal liver were transduced and injected into two recipient mice, and recipient mice were harvested at 3 weeks post-transplantation. From these recipient mice, peripheral blood was collected by retro-orbital bleed into EDTA containing tubes and the spleen, thymus, lymph nodes (inguinal, brachial, axillary), and bone marrow (both femora and tibiae) were harvested and processed into single cell suspensions and counted as described above. Red blood cells were removed from peripheral blood and spleen cell suspensions by incubation in red cell removal buffer (Supplementary Table S1) for 5 min on ice and then washed twice. Cell suspensions were incubated in combinations of fluorochrome-conjugated antibodies against suitable surface markers (Supplementary Table S2). Staining with Propidium iodide (PI) was used for dead cell exclusion and cells were analysed in a Fortessa1 flow cytometer (Becton Dickinson).

### Isolation of pre-leukaemic pre-B cells for RNA-sequence analysis

Pre-B cells were isolated from the bone marrow of recipient mice that had been transplanted 3 weeks earlier with *sgTfap4/Eµ-MYC/Cas9* or *sgControl/Eµ-MYC/Cas9* FLCs. B cells were enriched from bone marrow of all long bones by staining with a cocktail of biotinylated antibodies against TER119 (Ly76), MAC-1 (M1/70), GR-1 (RB6-8C5) for 20 min on ice, washed, then incubated with MagniSort Streptavidin Negative Selection Beads (Thermo Fisher Scientific) according to the manufacturer’s protocol to remove undesired erythroid and myeloid cells. The supernatant was transferred into a fresh tube, washed, and resuspended in 100 µl of a cocktail of fluorochrome-conjugated antibodies against B220 (RA3-6B2), IgM (5.1), c-KIT (2B8) and 1 × 10^6^ live (PI negative) pre-leukaemic pre-B cells (B220^+^ sIgM^−^ c-KIT^−^) were FACS sorted, centrifuged, and resuspended in QIAzol Lysis Reagent (Qiagen) and stored at −80 °C. Total RNA was extracted using the QIAgen miRNeasy Mini Kit according to the manufacturer’s instructions with optional on-column DNAase digestion. An input of 100 ng of total RNA was used to prepare mRNA libraries and indexed using the TruSeq RNA samples Prep Kit (illumina) according to the manufacturer’s protocol. The samples were sequenced on an Illumina NextSeq using paired-end sequencing. RNA-sequencing analysis are detailed in the Supplementary Material.

### Statistical analysis

Data are presented as mean ± standard error of the mean (SEM) unless otherwise stated in figure legends. Statistical analysis was performed using GraphPad Prism software (v9.4.0); differences between two groups was determined using Student’s *t* test; Welch’s correction was applied if data did not fit a normal model; ANOVA test was used for comparing multiple groups. In vitro cell death curves were all log transformed and fitted to a non-linear regression model and the IC50 derived using GraphPad Prism software (v9.4.0). Sample sizes were determined to be able to observe statistically significant differences between control mice/cells and the genetically modified mice/cells, and experiments were replicated at least three times.

## Results

### Deletion of TFAP4 in HSPCs accelerates c-MYC-driven lymphomagenesis

**Fig. 1:**
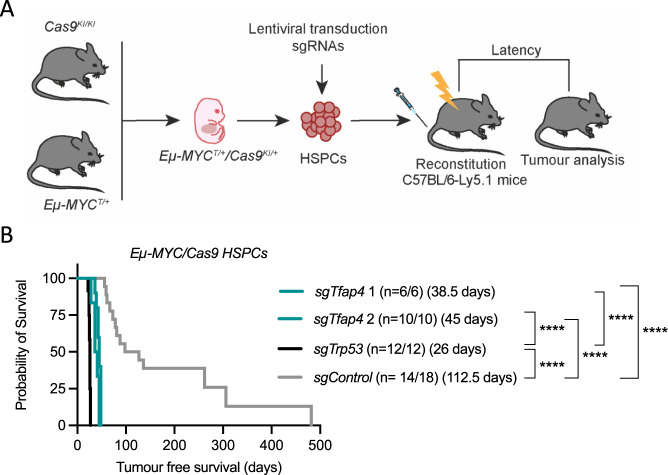
Loss of *Tfap4* accelerates c-MYC-driven lymphomagenesis. **A** Schematic representation of haematopoietic reconstitution of lethally irradiated recipient mice with donor fetal liver cells, a rich source of HSPCs. FLCs from doubly transgenic *Eµ-MYC/Cas9* E13.5 donor embryos were lentivirally transduced with vectors encoding BFP or CFP as a marker and sgRNAs targeting *Tfap4* (*sgTfap4*), a positive sgRNA targeting *Trp53* (*sgTrp53*) or a negative non-targeting control sgRNA (*sgControl*). These transduced donor FLCs were then transplanted into lethally irradiated C57BL/6-Ly5.1 recipient mice. Tumour-free survival of recipient mice was measured as days post-transplantation. Haematopoietic tissues and peripheral blood were harvested from tumour burdened recipient mice for further analysis. **B** Kaplan–Meier survival curve showing tumour-free survival of mice transplanted with either of two vectors containing a *sgTfap4*, a positive control *sgTrp53* or a negative *sgControl*. *n* indicates number of sick mice/number of mice transplanted with transduced HSPCs for each sgRNA. Median survival post-transplantation in days is indicated in brackets. ****p < 0.0001.

We set out to validate *Tfap4* as a top hit from a previous genome-wide CRISPR knockout screen [1] accelerating *Eµ-MYC* induced lymphomagenesis using independent sgRNAs targeting *Tfap4*. To this end, we isolated fetal liver cells (FLCs) (a rich source of HSPCs capable of reconstituting the haematopoietic system [26]) from *Eµ-MYC*/*Cas9* doubly transgenic day 13.5 embryos (E13.5) and independently transduced them with two sgRNAs targeting *Tfap4* (*sgTfap4*), a positive control sgRNA targeting *Trp53* (*sgTrp53*) [27] or a negative control sgRNA targeting human *BIM* or human *NLRC5* (*sgControl*). These sgRNA transduced *Eµ-MYC*/*Cas9* HSPCs were transplanted into lethally irradiated C57BL/6-Ly5.1 recipient mice that were then monitored for lymphoma development (Fig. 1A).

All recipient mice displayed characteristic *Eµ-MYC* lymphoma pathology, including enlarged lymph nodes, spleen and thymus, elevated white blood cell counts (WBC) and thrombocytopenia (Supplementary Fig. 1a, b). Mice transplanted with the *sgTfap4*/*Eµ-MYC/Cas9* HSPCs developed lymphoma at a slightly slower pace than mice that had been transplanted with *sgTrp53* transduced *Eµ-MYC/Cas9* HSPCs (median survival of *sgTfap4*-1 = 38.5 days; *sgTfap4*-2 = 45.5 days vs. *sgTrp53* = 26 days), but significantly faster than mice transplanted with *sgControl* transduced *Eµ-MYC/Cas9* HSPCs (112.5 days) (Fig. 1B). This finding validates that deletion of *Tfap4* markedly accelerates c-MYC-driven lymphomagenesis.

### TFAP4 deficient *Eµ-MYC* lymphomas are not selected for defects in the TRP53 pathway

**Fig. 2:**
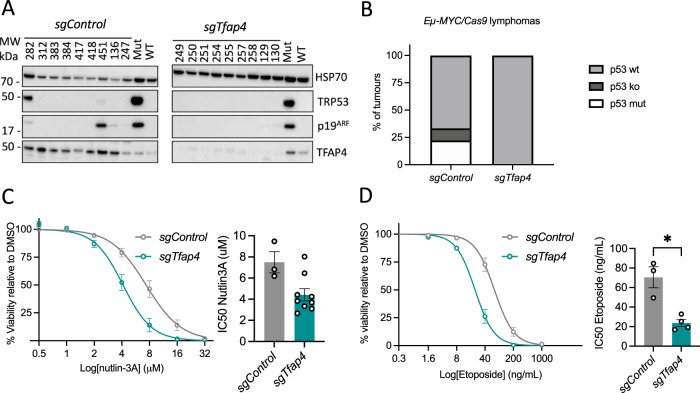
Deletion of *Tfap4* removes the selection pressure to acquire defects in the TRP53 pathway during c-MYC-driven lymphoma development. **A** Western blot analysis of *sgTfap4*/*Eµ-MYC/Cas9* and *sgControl*/*Eµ-MYC/Cas9* primary lymphomas for TRP53, p19/ARF, TFAP4 and HSP70 (protein loading control). The blot includes positive and negative control cell lysates for mutant (Mut) and wild-type (WT) TRP53. Molecular weight markers are indicated in kDa. **B** Summary graph representing percentages of *sgTfap4*/*Eµ-MYC/Cas9* (*n* = 9) and *sgControl*/*Eµ-MYC/Cas9* (*n* = 9) lymphomas that have defects in the TRP53 pathway as assessed by Western blotting for TRP53 and p19/ARF. TRP53 wt determined as lack of detectable protein expression of both TRP53 and p19/ARF. TRP53 knockout (ko) is determined as no detectable TRP53 protein expression with high expression of p19/ARF. Mutant TRP53 is identified by high level expression of both TRP53 and p19/ARF proteins. Cell viability response curves and corresponding IC50 graphs in *sgTfap4/Eµ-MYC/Cas9* and *sgControl/Eµ-MYC/Cas9* lymphoma cell lines 24 h after treatment with the indicated doses of nutlin-3A (**C**) or etoposide (**D**). Cell viability was determined by flow cytometry; live cells were identified as the Annexin V/PI double negative population. Data represent percentage mean survival of lymphoma cell lines at each dose (*sgControl* *n* = 3, *sgTfap4* *n* = 4–9). Data were log transformed and fitted to non-linear regression mean ± SEM. IC50 values were calculated using Prism Graphpad software. Each dot represents an independent lymphoma cell line; error bars represent mean ± SEM. Two-tailed Student’s *t* test, *p < 0.05.

Since ~25% of lymphomas that spontaneously develop in *Eµ-MYC* transgenic mice show defects in the TRP53 pathway, we examined the *sgTfap4*/*Eµ-MYC/Cas9* lymphomas, in which TFAP4 protein was absent, for abnormalities in the TRP53 pathway and compared them to the *sgControl/Eµ-MYC/Cas9* lymphomas (Fig. 2A). Interestingly, no TRP53 pathway alterations were detected in any of the *sgTfap4*/*Eµ-MYC/Cas9* lymphomas tested by Western blotting for the levels of TRP53 and p19/ARF proteins (high levels of these proteins are an indicator for mutations in the *Trp53* gene and/or TRP53 pathway defects) (Fig. 2A, B) [28]. This contrasts with the *sgControl/Eµ-MYC/Cas9* lymphoma cell lines, with ~30% of those exhibiting TRP53 pathway aberrations using this assay. To assess functionality of the TRP53 pathway, we generated cell lines from the primary *sgTfap4*/*Eµ-MYC/Cas9* and *sgControl*/*Eµ-MYC/Cas9* lymphomas and demonstrated by flow cytometry that they are readily killed by treatment with nutlin-3A (a drug that activates TRP53 by blocking its major negative regulator MDM2 [29]) (Fig. 2C), and the DNA-damaging chemotherapeutic drug etoposide (Fig. 2D) that can also kill lymphoma cells through activation of TRP53 [30]. Collectively, these data reveal that loss of TFAP4 reduces the selection pressure for *Eµ-MYC* lymphomas to acquire defects in the TRP53 tumour suppressor pathway.

### TFAP4 deficiency does not change reliance on pro-survival BCL-2 family members

Avoiding cell death is one of the hallmarks of cancer [31], and this is often achieved by abnormally increased expression of pro-survival BCL-2 family members [32]. *Eµ-MYC* lymphoma cells are generally dependent on the pro-survival BCL-2 family member MCL-1 for their sustained survival [24, 33]. We examined the responses of *sgTfap4*/*Eµ-MYC/Cas9* and *sgControl*/*Eµ-MYC/Cas9* lymphoma cell lines to the BH3 mimetic drugs ABT-199/Venetoclax [34] or S63845 [35] that inhibit BCL-2 or MCL-1, respectively. No differences in cell viability were observed between the *sgTfap4* vs. *sgControl/Eµ-MYC/Cas9* treated cells as measured at 24 h of treatment with these agents by flow cytometry (Supplementary Fig. 2a–c). These findings demonstrate that the absence of TFAP4 accelerates c-MYC-driven lymphoma development through mechanisms other than altering the dependency of these malignant cells on select pro-survival BCL-2 proteins.

### TFAP4 impairs differentiation of *Eµ-MYC* transgenic B lymphoid cells at the pre-B stage

**Fig. 3:**
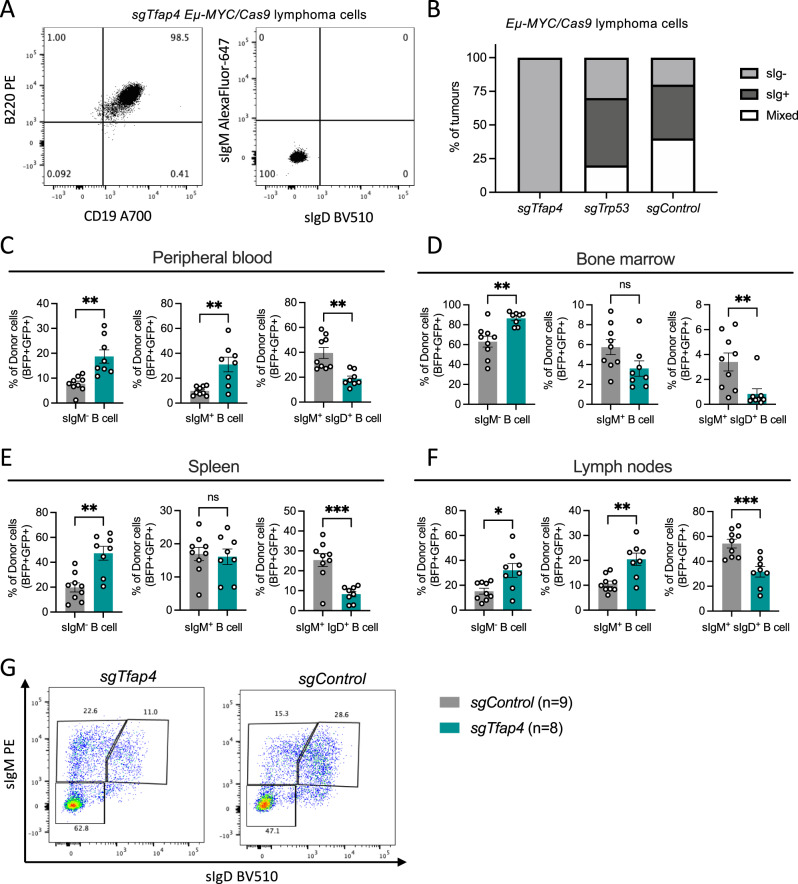
Deletion of TFAP4 in pre-leukaemic *Eµ-MYC* pre-B cells impairs B cell differentiation. **A** Representative flow cytometry plot of surface staining for B220, CD19, IgM and IgD of a *sgTfap4/Eµ-MYC/Cas9* lymphoma cell line. **B** Summary graph of surface Ig staining by flow cytometry of cell lines derived from *Eµ-MYC/Cas9* primary lymphomas of each genotype; *sgTfap4* (*n* = 10)*, sgTrp53* (*n* = 20) and *sgControl* (*n* = 5). sIg^−^ represents B220^+^ sIgM^−^ sIgD^−^ lymphomas; sIg^+^ represents B220^+^ sIgM^+^/sIgD^+^ lymphomas; and mixed indicates B220^+^ lymphoma cells with both sIgM^−^ and sIgM^+^ lymphoma cell populations. Lethally irradiated wild-type mice were reconstituted with *Eµ-MYC/*Cas9 FLCs transduced with either a *sgTfap4* or a *sgControl* vector. The haematopoietic cell subsets in these transplanted mice were analysed at 3 weeks post-transplantation by flow cytometry. The percentages of donor derived (GFP^+^ BFP^+^) B cell subsets; sIgM^−^ (B220^+^ sIgM/sIgD^−^), immature sIgM^+^ B cells (B220^+^ sIgM^+^ sIgD^−^) and mature sIgM^+^/sIgD^+^ B cells (B220^+^ sIgM/sIgD^+^) in the peripheral blood (**C**), bone marrow (**D**), spleen (**E**), and lymph nodes (**F**) of recipient mice were determined. **G** Representative flow cytometry dot plot to examine the different B cell subsets, gated on live donor derived lymphoid cells GFP^+^ BFP^+^ CD45.2^+^ B220^+^. Data presents mean ± SEM, each dot represents an individual recipient mouse that had been transplanted with *sgTFAP4/Eµ-MYC/Cas9* (*n* = 8) or *sgControl/Eµ-MYC/Cas9* (*n* = 9) FLCs. Unpaired two-tailed Student’s *t* test with Welch’s correction, *p < 0.05, **<0.01, ***<0.001.

Interestingly, we found that *sgTfap4/Eµ-MYC/Cas9* lymphoma cell lines all represented neoplastic counterparts of surface Ig negative pre-B cells. In contrast, the *sgControl* as well as *sgTrp53 Eµ-MYC/Cas9* lymphomas comprised a mixture of sIg negative pre-B lymphomas and sIg positive B cell lymphomas (Fig. 3A, B). This suggests that the absence of TFAP4 drives transformation at an early stage of B cell development. To better understand how TFAP4 deletion contributes to c-MYC-driven lymphoma development, a pre-leukaemic analysis was performed whereby recipient mice transplanted with either *sgTfap4* or *sgControl* transduced *Eµ-MYC/Cas9* HSPCs were harvested 3 weeks post-transplantation, a time point early during lymphomagenesis when their B lymphoid cells are not yet fully transformed. Bone marrow, spleen, thymus, lymph nodes and peripheral blood were analysed by flow cytometry to determine the proportions of the various B as well as T lymphocyte and myeloid cell subsets. No differences were observed in the total cellularity of the haematopoietic tissues between the *sgTfap4/Eµ-MYC/Cas9* vs. the *sgControl/Eµ-MYC/Cas9* groups (Supplementary Fig. 3a). Furthermore, the contribution of donor cells carrying both *Cas9* (GFP positive) and the sgRNA (BFP positive) was comparable between the *sgTfap4/Eµ-MYC/Cas9* vs. the *sgControl*/*Eµ-MYC/Cas9* HSPCs transplanted cohorts across all haematopoietic tissues examined (Supplementary Fig. 3b). However, these compartments were not completely composed of transduced donor HSPC derived cells. This can be explained because: (1) not all *Cas9-GFP* donor cells carried *sgRNA-BFP* due to the infection efficiency of the donor HSPCs; (2) not all host cells have been cleared from the haematopoietic tissues of recipient mice examined 3 weeks post-transplantation with donor cells. The latter is especially apparent for T lymphocytes and granulocytes: only a small percentage of these cell types were donor derived (Supplementary Fig. 3c, d). Thus, to assess the impact of *Tfap4* deletion in different cell types, only sgRNA/Cas9 doubly expressing (BFP^+^ GFP^+^) *Eµ-MYC* cells were examined.

Peripheral blood, bone marrow, spleen and lymph nodes showed significant differences in the B cell compartment between the *sgTfap4/Eµ-MYC/Cas9* vs. the *sgControl*/*Eµ-MYC/Cas9* HSPC transplanted cohorts. We detected higher proportions of surface Ig negative pro-B/pre-B cells (B220^+^ sIgM^−^ sIgD^−^) and a reduction in the proportions of mature B cells (B220^+^ sIg^+^) in the *sgTfap4/Eµ-MYC/Cas9* pre-leukaemic recipient mice compared to their *sgControl/Eµ-MYC/Cas9* counterparts (Fig. 3C–G). In the peripheral blood and lymph nodes an increase in the proportion of sIgM^+^ B cells was observed, likely because mature B cells circulate to these peripheral lymphoid tissues compared to the bone marrow and spleen, the sites of B cell development and maturation, respectively. This accumulation of pro-B/pre-B cells in the *sgTfap4/Eµ-MYC/Cas9* pre-leukaemic recipients is consistent with the immuno-phenotyping of the malignant lymphomas arising in these mice (Fig. 3A, B).

### TFAP4 deleted pre-leukaemic *Eµ-MYC* pre-B cells fail to upregulate transcriptional programmes required for B cell differentiation

**Fig. 4:**
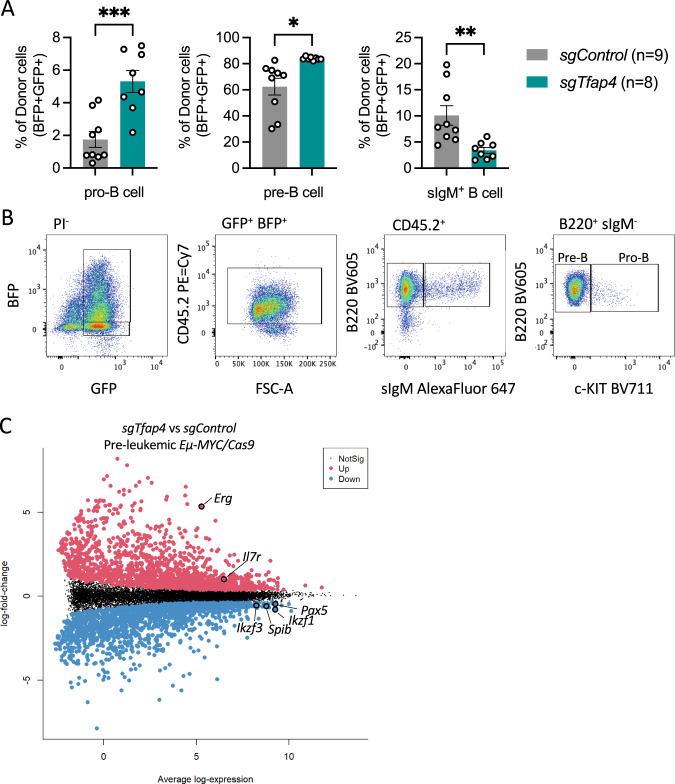
Pre-leukaemic *Eµ-MYC/Cas9* pre-B cells lacking TFAP4 are transcriptionally distinct from control *Eµ-MYC/Cas9* pre-B cells. Lethally irradiated recipient mice were reconstituted with *Eµ-MYC/Cas9* FLCs that had been transduced with either a vector encoding *sgTfap4* or a *sgControl*, and their pre-leukaemic cells were analysed at 3 weeks post-transplantation. **A** In the bone marrow, the percentages of pro-B cells, pre-B cells and immature B cells derived from donor (GFP^+^ BFP^+^) FLCs were determined by flow cytometric analysis. Data present mean ± SEM, Each dot represents an individual recipient mouse that had been transplanted with *sgTfap4* (*n* = 8) or *sgControl* (*n* = 9) transduced *Eµ-MYC/Cas9* FLCs. Unpaired two-tailed Student’s *t* test with Welch’s correction, *p < 0.05, **<0.01, ***<0.001. **B** Representative flow cytometry plots demonstrating gating strategy to identify the different donor derived (GFP^+^ BFP^+^ CD45.2^+^) B cell subsets in the bone marrow of recipient mice: pro-B (B220^+^ sIgM^−^ c-KIT^+^), pre-B (B220^+^ sIgM^−^ c-KIT^−^), immature B (B220^+^ sIgM^+^) cells. **C** Mean difference plot presenting log-fold changes (average of each group) with significantly differentially expressed genes highlighted (FDR < 0.05), red = up-regulated, blue = down-regulated. Some genes of interest are labelled from RNA-seq analysis of donor derived pre-leukaemic *Eµ-MYC/Cas9* pre-B cells (GFP^+^ BFP^+^ B220^+^ sIgM^−^ c-KIT^−^) from *sgTfap4/Eµ-MYC/Cas9* (*n* = 8) or *sgControl/Eµ-MYC/Cas9* (*n* = 6) cohorts isolated from the bone marrow of recipient mice.

As TFAP4 is a transcriptional regulator, we next sought to understand how its loss might alter cell signalling pathways to accelerate c-MYC-driven lymphoma development. To this end we examined the B cell subsets in the bone marrow, the site of B cell development, by flow cytometry in the pre-leukaemic recipient mice. We observed a significant reduction in the percentages of donor derived mature B cells (B220^+^ sIgM^+^ sIgD^+^) and an increase in pro-B (B220^+^ sIgM^−^ cKIT^+^) and pre-B cells (B220^+^ sIgM^−^ cKIT^−^) in the *sgTfap4*/*Eµ-MYC*/Cas9 recipient mice compared to the *sgControl/Eµ-MYC*/Cas9 control recipient mice (Fig. 4A).

We isolated donor derived pre-leukaemic pre-B cells (GFP^+^ BFP^+^ B220^+^ sIgM^−^ cKIT^−^) from both the sg*Tfap4/Eµ-MYC/Cas9* recipient mice and the *sgControl/Eµ-MYC*/Cas9 control counterparts (Fig. 4B) and analysed their transcriptional programme by RNA-sequencing. Since fully transformed *Eµ-MYC* lymphomas are monoclonal they express only one Ig species. Our analysis revealed that the pre-leukaemic *sgTfap4/Eµ-MYC/Cas9* and *sgControl/Eµ-MYC/Cas9* pre-B cells expressed a variety of Ig transcripts, confirming that the cells we analysed were not fully transformed (Supplementary Fig. 4a). The absence of TFAP4 markedly altered the transcriptional profile of the pre-leukaemic *Eµ-MYC* pre-B cells (Supplementary Fig. 4b). A total of 3218 genes were differentially expressed between the *sgTfap4/Eµ-MYC/Cas9* vs. *sgControl/Eµ-MYC/Cas9* pre-leukaemic pre-B cells (Fig. 4C). Further analysis revealed many differentially expressed hallmark gene set pathways, such as the MYC (regulates TFAP4 [9]) or TRP53 (indirectly regulated by TFAP4 in human cells [36, 37]) pathways (Supplementary Fig. 4c).

**Fig. 5:**
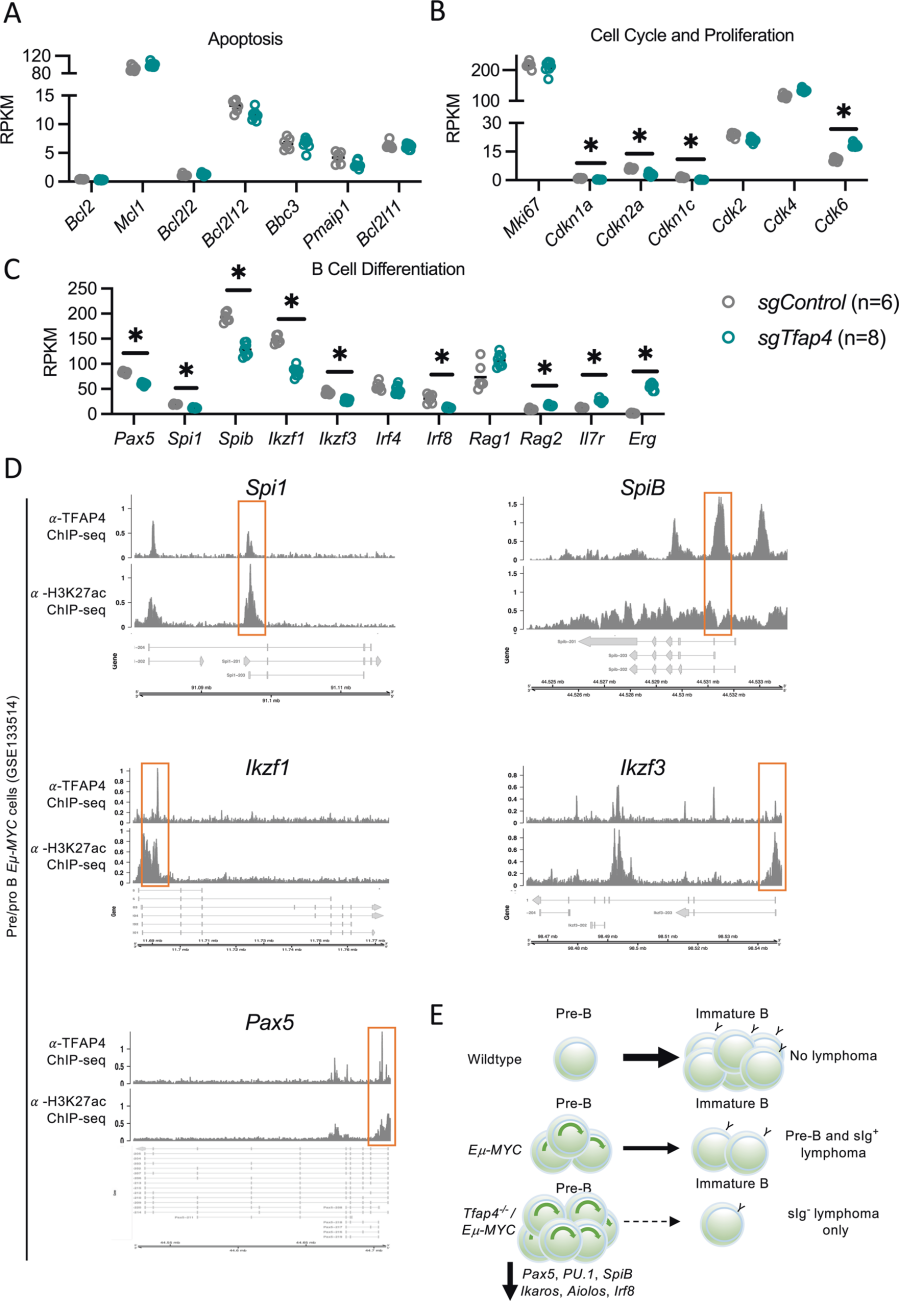
Genes regulating B cell differentiation are down-regulated in TFAP4 deleted pre-leukaemic *Eµ-MYC* pre-B cells. Lethally irradiated recipient mice were transplanted with *Eµ-MYC/Cas9* FLCs that had been transduced with a vector containing either a *sgTfap4* (*n* = 8) or a *sgControl* (*n* = 6), and the pre-leukaemic pre-B cells from these recipient mice were analysed at 3 weeks post-transplantation by RNA-sequencing. Relative expression (RPKM—reads per kilobase million) of selected gene transcripts regulating apoptosis (**A**), cell cycling and proliferation (**B**), or master transcription factors and coordinators of B cell differentiation as well as *Erg* (**C**). Statistical significance *adj. *p* < 0.05, FDR < 0.05. **D** *Spi1*, *SpiB*, *Ikzf1, Ikzf3* and *Pax5* genomic loci coverage plots of anti-TFAP4 and anti-H3K27ac (a marker of accessible chromatin) binding in pre-leukaemic *Eµ-MYC* pro/pre-B cells. *Y*-axis represents counts per million (CPM), gene isoforms pictured, thick bar indicates exons, orange box highlights binding. Data accessed from GSE133514 [20]. **E** Proposed mechanism: in wild-type mice normal B cell development occurs, whereby pre-B cells differentiate into immature B cells expressing surface IgM. In *Eµ-MYC* transgenic mice, c-MYC is abnormally overexpressed in the B cell lineage causing excess proliferation of pro-B/pre-B cells and a partial block in their differentiation, thereby producing an abnormally expanded pool of pre-leukaemic pre-B cells and some sIg^+^ B cells. Some of these cells will acquire oncogenic mutations that can collaborate with c-MYC over-expression in neoplastic transformation and consequently give rise to pre-B or B cell lymphoma. The absence of TFAP4 in the *Eµ-MYC* setting results in an even larger pool of pre-leukaemic pre-B cells arising due to the further restriction of differentiation by downregulation of transcription factors that are critical for B cell differentiation. This even larger pool of highly proliferative pre-leukaemic pre-B cells is thus more likely to acquire additional mutations that drive transformation into malignant surface Ig^−^ pre-B cell lymphoma.

To elucidate the contribution of loss of TFAP4 to c-MYC-driven lymphoma development we compared the expression of genes within certain hallmarks of cancer pathways that TFAP4 has been reported to regulate. Consistent with the in vitro cell death assays using BH3 mimetic drugs targeting BCL-2 or MCL-1 in malignant *Eµ-MYC* lymphoma cells, we observed no differences in the expression of any of the BCL-2 family member genes (Fig. 5A). Mutations in TFAP4 have been implicated in abnormal cell proliferation in certain solid cancers [8]. We observed no differences in the expression of *Mki67* (aka Ki67), a marker of cell division, and only a minor reduction of cell cycle genes (*Cdkn1, Cdkn2a, Cdkn1c*) (these are all known target genes of both MYC and TFAP4 [38, 39]) between the *sgTfap4/Eµ-MYC/Cas9* vs. the *sgControl/Eµ-MYC/Cas9* pre-leukaemic pre-B cells (Fig. 5B). This indicates that increased cell proliferation is not the mechanism by which loss of TFAP4 accelerates c-MYC-driven lymphoma development.

Our pre-leukaemic analysis and the immuno-phenotyping of the malignant *Eµ-MYC* lymphoma cells revealed that the absence of TFAP4 resulted in the accumulation of pre-B cells, indicating a block in B cell development as a possible cause of accelerated c-MYC-driven lymphomagenesis. Therefore, we assessed the expression of genes encoding master transcriptional regulators of B cell differentiation. Interestingly, sg*Tfap4/Eµ-MYC/Cas9* pre-leukaemic pre-B cells expressed lower levels of *Pax5*, the master regulator of B cell lineage specification (Fig. 5C). Furthermore, we observed a reduction in *Spi1* (*PU.1*), *SpiB, Irf8, Ikzf1 (Ikaros*) and *Ikzf3* (*Aiolos*), genes that orchestrate differentiation of common lymphoid progenitors into pro-B, pre-B and immature B cells (Fig. 5C) [40]. Interestingly, a previously reported TFAP4 ChIP-sequencing data set from pre-leukaemic *Eµ-MYC* pro-/pre-B cells [20] demonstrated direct binding of TFAP4 to the promoters of the *Ikzf1, Ikzf3, Spi1, SpiB* and *Pax5* genes (Fig. 5D). Notably, these genes (data not available for Spi1) are also direct MYC target genes [41]. These findings demonstrate that the absence of TFAP4 impairs the differentiation of highly proliferative *Eµ-MYC* pre-leukaemic pre-B cells by reducing the levels of transcription factors that are critical for B cell differentiation (Fig. 5E).

## Discussion

*Tfap4* is a direct c-MYC target gene that was reported to contribute to certain c-MYC regulated cellular processes, including apoptosis, cell proliferation and cellular senescence [42]. Induction of *Tfap4* by c-MYC to coordinate these processes has been implicated in neoplastic transformation of several solid cancers [8]. This contrasts our findings, where not over-expression but rather the absence of TFAP4, through CRISPR/Cas9 mediated deletion, accelerated c-MYC-driven lymphomagenesis. In the context of c-MYC-driven lymphoma development, loss of TFAP4 does not alter apoptosis or cell proliferation, as the genes regulating these processes were not differentially expressed in pre-leukaemic pre-B cells lacking TFAP4. However, we observed aberrantly reduced expression of transcription factors that are critical for B cell differentiation, including *Ikzf1, Ikzf3, Spi1, SpiB* and *Pax5*. This indicates that TFAP4 deficiency accelerates lymphoma development by impairing B cell differentiation, representing an emerging hallmark of cancer [31]. Of note, *Tfap4* deletion on its own either in the whole organism or specifically in B lymphoid cells does not drive tumorigenesis. [9, 20]. Preliminary experiments deleting TFAP4 in HSPCs and transplanting them into lethally irradiated animals similarly did not cause any malignant growth (our data not shown).

Recently, Tonc et al. [20] showed that *TFAP4* is frequently mutated in human mature B cell malignancies. Furthermore, they showed, that patients with paediatric B progenitor cell acute lymphoblastic leukaemia (B-ALL) showing *TFAP4*-low/*MYC*-high expression had significantly poorer overall survival compared to those showing *TFAP4*-high*/MYC*-high expression. Additionally, this study revealed that loss of *Tfap4*, either a single allele or both alleles, accelerated c-MYC-driven lymphoma development in mice. This work was conducted by employing both whole body *Tfap4* knockout as well as conditional *Tfap4* knockout mouse models crossed with *Eµ-MYC* transgenic mice as well as with other mouse models of c-MYC driven lymphoid malignancies. Concordant with our findings, all tumours Tonc et al. found in their *Tfap4*^*−/−*^ *Eµ-MYC* mice were pro-B/pre-B cell lymphomas. However, their pre-leukaemic analysis using *Tfap4*^*+/−*^ *Eµ-MYC* mice showed no differences in the numbers of cells within the different B cell subsets [20]. The difference between their findings and our observations is likely due to the different model systems employed. In our case, the use of CRISPR/Cas9 results in complete deletion of *Tfap4*, evident by loss of TFAP4 protein (Fig. 2A), whereas the pre-leukaemic cells Tonc et al. examined retained one allele of *Tfap4*, albeit some of these cells selected for loss of the second allele, highlighting that there is potent selection for loss of *Tfap4* in c-MYC-driven lymphoma development. Despite not observing differences in the proportions of B cell subsets at the different stages of differentiation in their pre-leukaemic mice, Tonc et al. [20] concluded that TFAP4 restricts c-MYC regulated B cell stemness because they found an upregulation of *Erg*, a direct target gene of TFAP4, that regulates haematopoietic stemness. While we also observed increased levels of *Erg* in the *sgTfap4/Eµ-MYC/Cas9* pre-leukaemic pre-B cells in our RNA-seq analysis (Fig. 5C), we noted a reduction in several master regulators of B cell differentiation. We observed abnormally reduced expression of *Spi1* (*PU.1*), *SpiB*, *Ikzf1* (*Ikaros*) and *Ikzf3* (*Aiolos*) in *sgTfap4/Eµ-MYC/Cas9* pre-leukaemic pre-B cells; these are direct target genes of both TFAP4 and MYC [41]. The contribution of these genes to normal B cell development and the impact of defects in their expression have been well characterised (reviewed by Pang et al. [40]). Interestingly, the deletion of *Spi1*  (*PU.1*) or *SpiB* impairs B cell differentiation, and mice lacking these transcriptional regulators develop B-ALL [43, 44]. Furthermore, loss of *Ikzf11* (*Ikaros*) or *Ikzf3* (*Aiolos*) is associated with B-ALL development in both mice and humans [40, 45, 46]. *Pax5* deletion has been shown to prevent B cell differentiation beyond the pro-B cell stage [40] and its knockdown drives the development of B-ALL due to a differentiation block [47]. We therefore propose that TFAP4 is required for differentiation of c-MYC over-expressing pre-B cells through transcriptional regulation of *Spi1*, *SpiB*, *Ikzf1, Ikzf3* and *Pax5*. The reduced activation of these genes caused by the absence of TFAP4, in combination with aberrant c-MYC expression, prevents normal B cell differentiation causing an increase in the pool of highly proliferative pro-B/pre-B cells facilitating the acquisition of oncogenic lesions that cooperate with deregulated c-MYC expression in lymphomagenesis (Fig. 5E). Thus, restoring TFAP4 expression in c-MYC driven blood cancers, might ameliorate disease progression, not by killing the tumour cells, but by inducing normal differentiation of the self-renewing pre-B lymphoma cell pool into less proliferative immature/mature B cells, albeit only if these cells are dependent on sustained absence of TFAP4 for lymphoma maintenance and not only for lymphoma development.

## Supplementary information

Supplementary Figures and Legends

Supplementary Methods and Tables

Checklist

Supplementary File

## Data Availability

RNA-sequencing data have been deposited on NCBI-GEP with the accession number GSE225684.
