# Supplementary material for: Deletion of the transcriptional regulator TFAP4 accelerates c-MYC-driven lymphomagenesis
Source: Cell Death Differ. 2023 Mar 9;30(6):1447–56. doi: 10.1038/s41418-023-01145-w (PMC10244435; doi:10.1038/s41418-023-01145-w)
Supplement: Supplementary file 1 — Supplementary Figures and Legends [file 41418_2023_1145_MOESM1_ESM.pdf]

Supplementary Figure 1

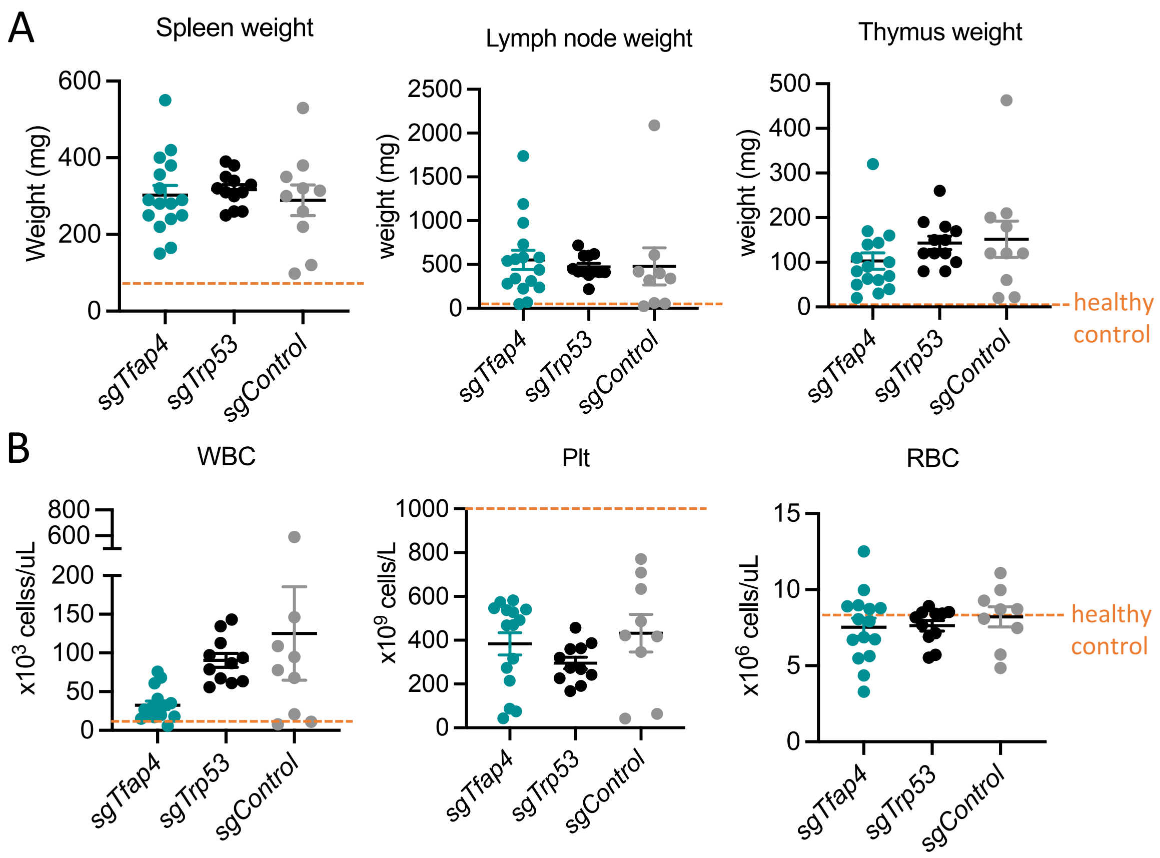

Supplementary Figure 1.

A, Weights of spleen, thymus, lymph nodes (combined axillary, brachial, inguinal) of lymphoma burdened recipient mice that had been transplanted with *sgTfap4* (n=16)*, sgTrp53* (n=12) or *sgControl* (n=10) transduced *Eμ-MYC/Cas9* FLCs. **B,** Number of white blood cells (WBC), platelets (Plt) and red blood cells (RBC) in peripheral blood of lymphoma burdened recipient mice that had been transplanted with *sgTfap4* (n=16)*, sgTrp53* (n=12) or *sgControl* (n=10) transduced *Eμ-MYC/Cas9* FLCs, as enumerated using the ADVIA. Organ weights and peripheral blood counts from healthy 7-week-old unmanipulated C57BL/6 mice are indicated by the orange dotted line. Each dot represents an individual mouse, error bars represent ± SEM**.**

Supplementary Figure 2

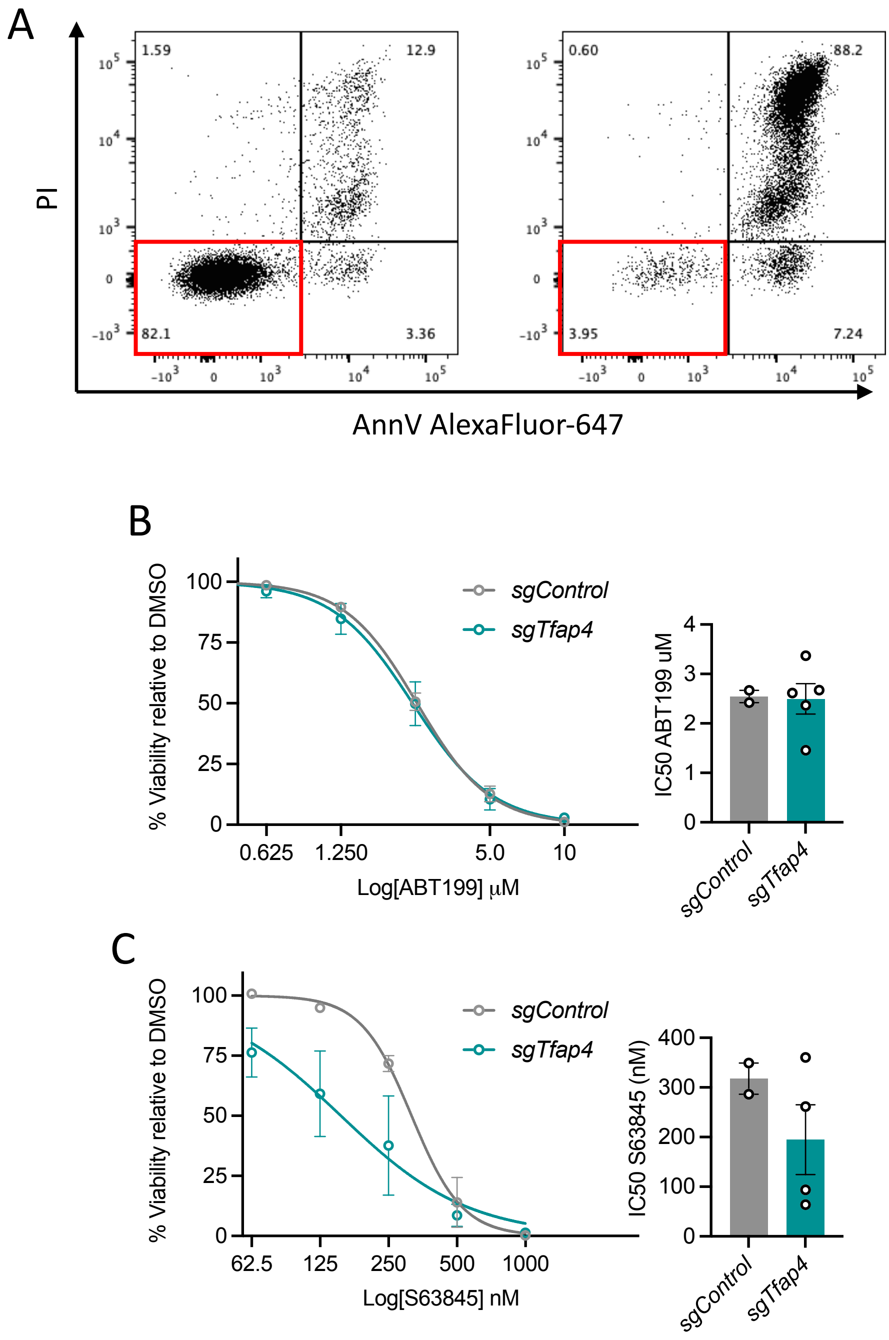

Supplementary Figure 2

A, Representative flow cytometry dot plots of Annexin-V plus PI staining to measure cell viability following *in vitro* drug treatment of the indicated lymphoma cells; live cells are the Annexin-V/PI double negative cell population (red box). **B-C,** Cell survival data and corresponding IC50 graphs for *sgTfap4/ Eμ-MYC/Cas9* and *sgControl/Eμ-MYC/Cas9* lymphoma cell lines 24 h after treatment with the indicated doses of the BH3 mimetic drugs ABT-199/Venetoclax (inhibitor of BCL-2) **(B)** or S63845 (inhibitor of MCL-1) **(C)**. Cell viability was determined by flow cytometry; live cells are the Annexin-V/PI double negative cell population. Data represent percentage mean survival of lymphoma cell lines at each dose (*sgControl* n=2, *sgTfap4* n=4). Data were log transformed and fitted to non-linear regression mean ± SEM. IC50 values were calculated using Prism Graphpad software. Each dot represents an independent lymphoma cell line; error bars represent ± SEM. Two-tailed Student *t*-test, $^{*}P<0.05$.

Supplementary Figure 3

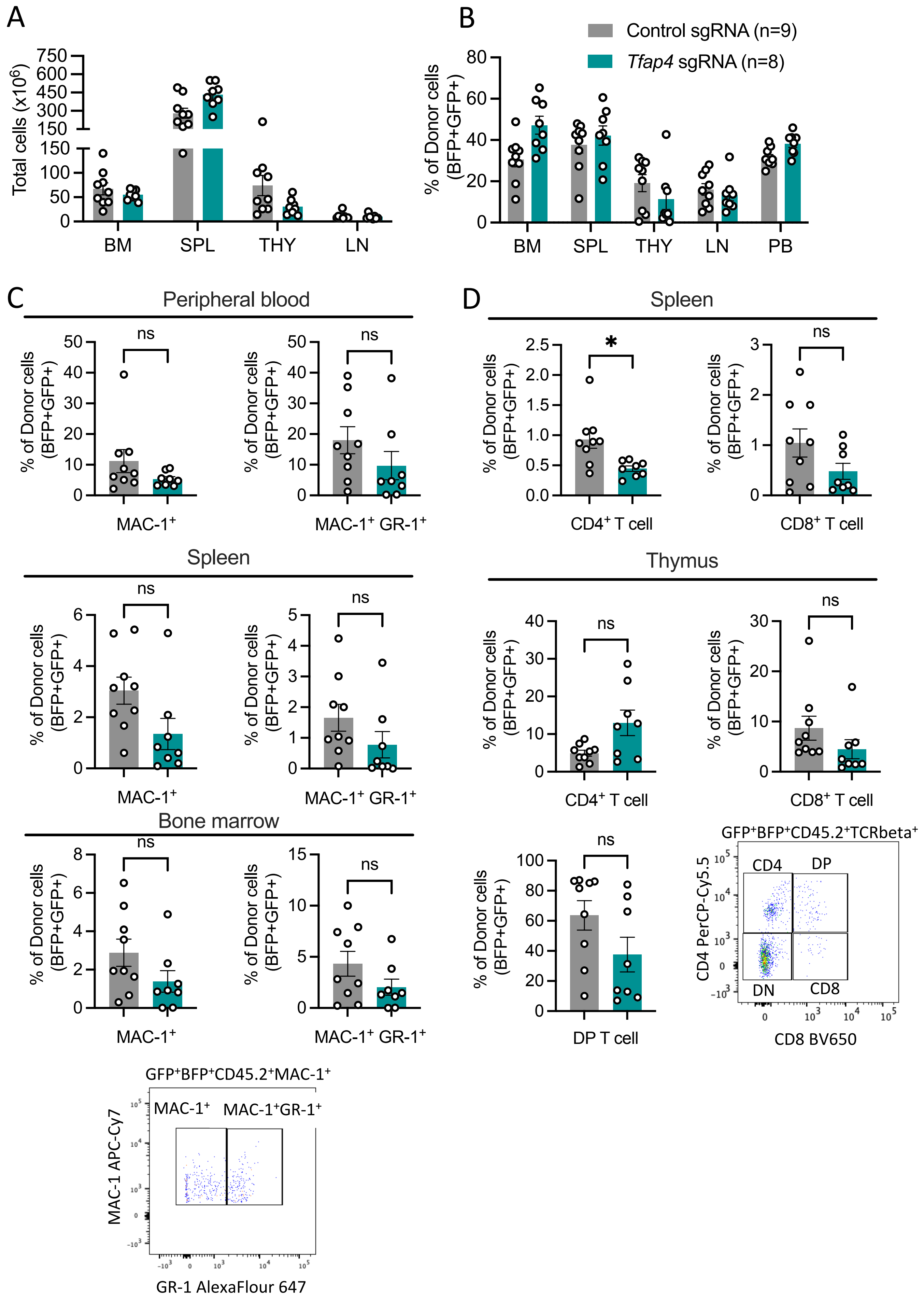

Supplementary Figure 3

Lethally irradiated wild-type mice were transplanted with *Eμ-MYC/*Cas9 FLCs that had been transduced with a vector containing either a *sgTfap4* or a *sgControl*. In these recipient mice, the composition of haematopoietic cell subsets across the indicated tissues was analysed at 3 weeks post-transplantation by flow cytometry. **A,** Total cellularity of haematopoietic tissues and **B,** proportions of donor derived cells carrying the sgRNA (GFP^+ BFP^+) for each haematopoietic tissue (bone marrow - BM, spleen - SPL, thymus - THY, lymph nodes – LN) in *sgTfap4/Eμ-MYC/Cas9* or *sgControl/Eμ-MYC/Cas9* pre-leukaemic recipient mice. **C,** Percentages of donor derived (GFP^+ BFP^+) macrophages/monocytes (MAC-1^+GR-1^-) and neutrophils (MAC-1^+GR-1^+) from pre-leukaemic recipient mice that had been transplanted with *sgTfap4/Eμ-MYC/Cas9* or *sgControl/Eμ-MYC/Cas9* FLCs in the peripheral blood, spleen and bone marrow. Representative flow cytometry plot of spleen cells gated on live GFP^+ BFP^+ $CD45.2^+$ MAC-1^+ cells. **D,** Percentages of donor derived (GFP^+ BFP^+) $CD4^+$ or $CD8^+$ T cells in the spleen or $CD4^+$, $CD8^+$ or $CD4^+$ $CD8^+$ double positive (DP) T lymphoid cells in the thymus. Representative flow cytometry plot of a thymus for T lymphoid cells gated on live GFP^+ BFP^+ $CD45.2^+$ $TCR\beta^+$ cells. *sgTfap4* (n=8), *sgControl* (n=9). Data represents mean ± SEM, each dot represents an individual mouse *sgTFAP4* (n = 8), *sgControl* (n = 9). Unpaired two-tailed Student's *t*-test with Welch's correction, 'ns' not significant.

Supplementary Figure 4

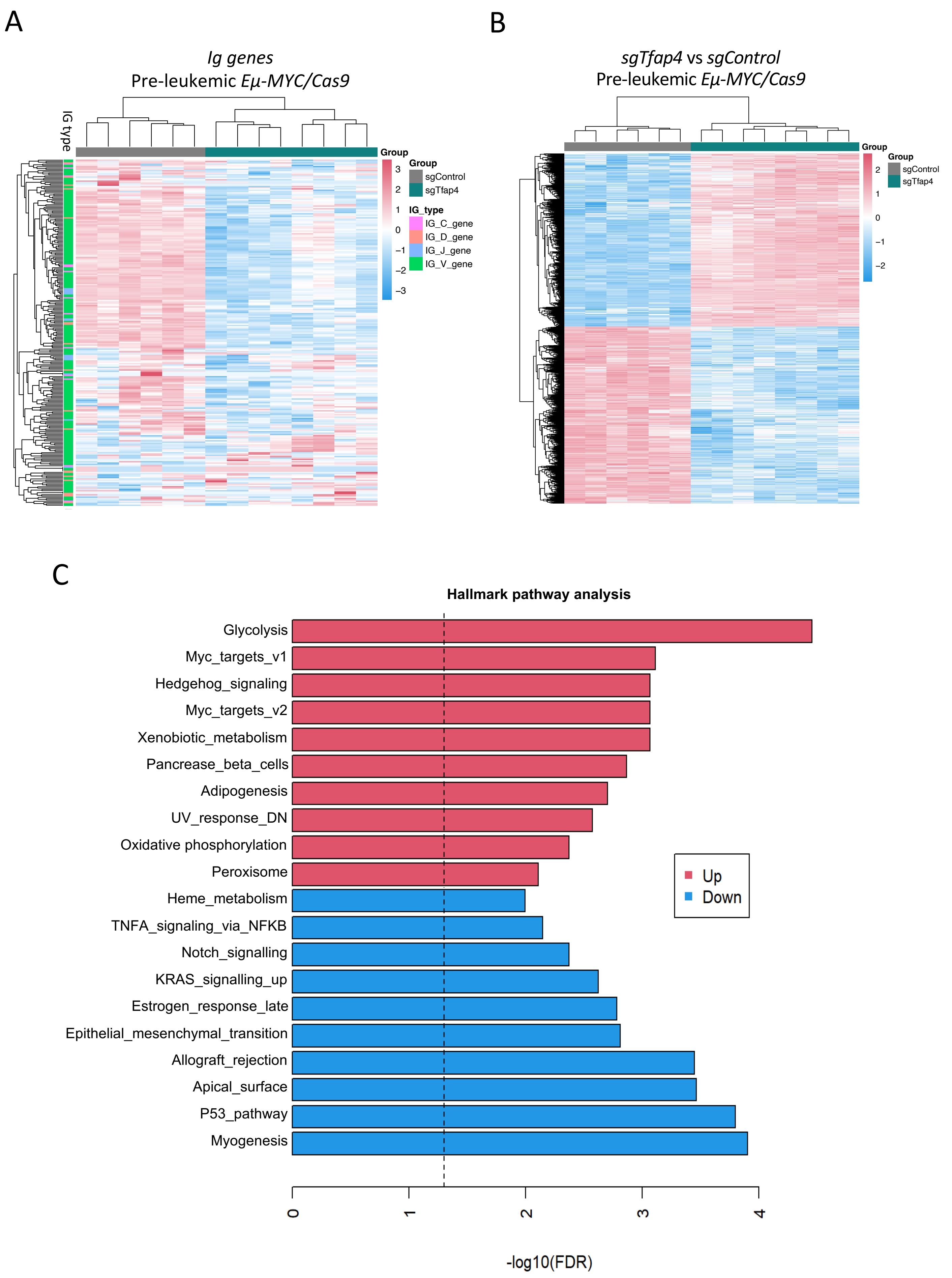

Supplementary Figure 4

A-C, RNA-seq analysis of donor derived pre-leukaemic *Eμ-MYC/Cas9* pre-B cells (GFP^+ BFP^+ $B220^+$ $sIgM^-$ $c\text{-}KIT^-$) from *sgTfap4/Eμ-MYC/Cas9 (*n=8) or *sgControl/Eμ-MYC/Cas9* (n=6) cohorts isolated from the bone marrow of recipient mice. **A,** Heatmap representation of Ig genes in pre-leukaemic pre-B cells of the indicated genotypes demonstrating variability in these transcripts within each sample and between different groups. **B,** Heatmap showing differential gene expression between the pre-leukaemic *sgTfap4l/Eμ-MYC/Cas9* pre-B cells and the *sgControl/Eμ-MYC/Cas9* control pre-leukaemic pre-B cells. **C,** Hallmark gene set pathway analysis showing the top 10 differentially (up and down) expressed pathways between pre-leukaemic *sgTfap4/Eμ-MYC/Cas9* pre-B cells *vs* *sgControl/Eμ-MYC/Cas9* pre-leukaemic pre-B cells.
