# Supplementary material for: Deletion of the transcriptional regulator TFAP4 accelerates c-MYC-driven lymphomagenesis
Source: Cell Death Differ. 2023 Mar 9;30(6):1447–56. doi: 10.1038/s41418-023-01145-w (PMC10244435; doi:10.1038/s41418-023-01145-w)
Supplement: Supplementary file 2 — Supplementary Methods and Tables [file 41418_2023_1145_MOESM2_ESM.docx]

**Supplementary Table S1**

Detailed recipes for media and buffers

| Media/buffer | Recipe |
| --- | --- |
| HEK293T medium | DMEM (Gibco) medium containing 10% (v/v) heat inactivated foetal calf serum (HI-FCS; Gibco), 100 U/mL Penicillin (Sigma) and 100 µg/mL streptomycin (Sigma) |
| FMA medium | DMEM (Gibco) supplemented with 10% (v/v) HI-FCS (Gibco), 50 µM B-mercaptoethanol (Sigma-Aldrich), 23.8 mM sodium bicarbonate (Merck), 1 mM HEPES (WEHI Media Kitchen), 13.5 µM folic acid (Sigma), 0.24 mM L-Asparagine monohydrate (Sigma), 0.55 mM L-Arginine monohydrochloride (Sigma), 22.2 mM D-glucose (Ajax), 100 U/mL Penicillin (Sigma) and 100 µg/mL streptomycin (Sigma) |
| Foetal liver medium | Alpha-minimum essential medium (alpha-MEM) Glutamax (Gibco) containing 10% FCS, 1x Glutamax, 1 mM sodium pyruvate, 100 U/mL penicillin, 100 µg/mL streptomycin, 10 mM HEPES, 50 µM B-mercaptoethanol that was further supplemented with recombinant cytokines 10 ng/mL IL-6, 100 ng/mL mouse stem cell factor (SCF), 50 ng/mL thrombopoietin (TPO) and 10 ng/mL FLT-3 ligand kindly provided by Dr. Jian-Guo Zhang (WEHI) |
| RIPA buffer | 50 mM Tris-HCl, 150 mM NaCl, 1% NP-40, 0.5% DOC, 0.1% SDS |
| Laemmli buffer | 0.25 M Tris.HCl pH 6.8, 40% glycerol, 0.8% SDS, 0.1% bromophenol blue, 10% B-mercaptoethanol |
| MES running buffer | 50 mM MES - 4 morpholine ethane sulfonic acid, 50 mM Tris base, 1mM EDTA, 0.1% w/v SDS |
| TAE buffer | 40 mM Tris Acetate, 1 mM EDTA pH 8.0 |
| FACS buffer | PBS supplemented with 5% (v/v) FCS and 5 µM EDTA (pH 8.0) |
| PBS-T | PBS, 0.1% (v/v) Tween20 |
| Red cell removal buffer | 156 mM Ammonium Chloride, 11.9 mM Sodium Bicarbonate, 0.097mM EDTA |

**Supplementary Table S2**

Table of antibodies used for surface staining by flow cytometry

| Marker detected | Antibody Clone |
| --- | --- |
| B220 | RA3-6B2 |
| IgM | 5.1 |
| IgD | 11-26C |
| CD19 | ID3 |
| T cell receptor beta (TCRβ) | H57-597 |
| CD4 | GK1.5 |
| CD8 | 53.6.7 |
| MAC1 | M1/70 |
| GR-1 | RB6-8C5 |
| CD45.2 | S450-15-2 |
| c-KIT | 2B8 |

**Supplementary Table S3**

Table of Western blotting primary and secondary antibodies

| Antibody | Clone | Supplier |
| --- | --- | --- |
| Primary antibodies | | |
| mouse-anti-TRP53 | CM5 | Novocastra^TM^ Leica Biosystems |
| mouse-anti-p19/ARF | 5.C3.1 | Rockland |
| mouse-anti-AP4 | A-8 | Santa Cruz Biotechnology, INC. |
| mouse-anti-HSP70 (loading control) | N-6 | a gift from Dr. R. Anderson, Olivia Newton John Centre, Melbourne, Australia |
| Secondary antibodies | | |
| goat-anti-mouse IgG conjugated to HRP |  | Southern Biotech |
| goat-anti-rat IgG conjugated to HRP |  | Southern Biotech |
| goat-anti-rabbit IgG conjugated to HRP |  | Southern Biotech |

**Supplementary Table S4**

Next Generation Sequencing target site primers

| sg*Tfap4* 1 target site primers | FWD 5’-GTGACCTATGAACTCAGGAGTCCATTCCACTGACCCCCGAGAC  REV 5’- CTGAGACTTGCACATCGCAGCCTTCTCCTGTTCAAGAGAGAA |
| --- | --- |
| sg*Tfap4* 2 target site primers | FWD 5’ GTGACCTATGAACTCAGGAGTCCCTGTTCTCCCTGGCAGCCTA  REV 5’- CTGAGACTTGCACATCGCAGCATTCTGCTGTCTGCTGGAGAA |

**sgRNA lentiviral vectors**

Constitutively expressed sgRNAs targeting *Tfap4* (sgRNA-1 5’-CGCATGCAGAGTATCAACGCGG; sgRNA-2 5’GATTGCCAACAGCAACGAGCGG) and a negative control sgRNA targeting *NLRC5* (sgRNA-1 5’- GCTGCAGAAGTGTCAGCTCCAGG) in a vector also containing a constitutively expressed BFP tag were obtained from the Merck CRISPR glycerol stock arrayed whole mouse genome sgRNA library, available at WEHI ([www.sigmaaldrich.com](http://www.sigmaaldrich.com) MSANGERG)*.* A positive control sgRNA targeting *Trp53* (5’-GGCAACTATGGCTTCCACCT); and a negative control sgRNA targeting human *BIM* (5’-GCCCAAGAGTTGCGGCGTAT), both in a vector containing a CFP tag, were derived from the constitutive sgRNA FUGW expression vector previously described ^1^ and used for haematopoietic reconstitution experiments.

**Lymphoma analysis**

Sick HSPC transplanted mice were euthanised and peripheral blood was collected and analysed using an ADVIA haematology analyser (Bayer). Haematopoietic tissues (spleen, thymus, lymph nodes – inguinal, axillary, brachial - and bone marrow from both femora and tibiae) were harvested and mashed through 100 µM filters to generate single cell suspensions in FACS buffer (Supplementary Table S1) and total cellularity was determined using a TC20 automated cell counter (Bio-Rad). Pellets of 2-5x10^6^ cells were collected for DNA and protein analysis. Lymphoma cell lines were derived by plating serial dilutions of tumour cells from enlarged haematopoietic tissues in FMA medium (described above). Lymphoma cell lines were immunophenotyped by staining with fluorochrome conjugated antibodies (Supplementary Table S2) in FACS buffer supplemented with Fc receptor block (2.4G2 hybridoma supernatant, WEHI). Donor cells were identified as CD45.2 positive, Cas9-eGFP and sgRNA CFP or BFP positive. Dead cells were excluded by gating on propidium iodide (PI) negative cells and live cells analysed in a Fortessax20 flow cytometer (Becton Dickinson). Data were analysed with FlowJo^TM^ analysis software.

**Genotyping**

Total DNA was extracted from ear-clips of adult mice or tails of day (E) 13.5 embryos using tail lysis buffer (Viagen Biotech) and proteinase K. DNA (1 µL) was PCR amplified in a mastermix of GoTaq Green (Promega) containing specific primers at a final concentration of 0.5 pmol/µL using the cycling conditions: 94°C for 4 min followed by 30 cycles of (94°C for 40 sec, 55°C for 30 sec, 72°C for 60 sec) and finally 72°C for 5 min. PCR products were size separated by gel electrophoresis on a gel composed of 2% DNA grade agarose (Bioline) in TAE buffer (Supplementary Table S1) containing ethidium bromide (0.2 µg/mL, Sigma) and imaged on GelDoc DOCTM XR+ Gel documentation system (Bio-Rad).

*Eµ-MYC*: ~900 bp

*MYC-1*: 5’ -CAGCTGGCGTAATAGCGAAGAG

*MYC-2*: 5’ -CTGTGACTGGTGAGTACTCAACC

**Western blotting**

Total protein was isolated from cell pellets of lymphoma bearing tissues by lysis in RIPA buffer (Supplementary Table S1) supplemented with complete protease inhibitor cocktail (Roche). Protein concentration was determined using the Bradford Assay (Bio-Rad), 20 µg of protein sample was prepared in Laemmli buffer (Supplementary Table S1), denatured at 100°C for 5 min, and size fractionated by gel electrophoresis on 4-12% NuPAGE^TM^ Bis-Tris polyacrylamide gels (Thermo Fisher Scientific) in MES Running Buffer (Supplementary Table S1). Proteins were then transferred onto nitrocellulose membranes (Life Technologies) using the iBlot membrane transfer system (Thermo Fisher Scientific) and membranes were blocked in PBS-T (Supplementary Table S1) and 5% (w/v) skim milk. The primary and secondary antibodies (Supplementary Table S3) were diluted in PBS-T and 5% (w/v) skim milk. Luminata Forte Western horseradish peroxidase (HRP) substrate (Merck Millipore) was added for imaging of protein bands using the ChemiDoc Imaging System with ImageLab software (Bio-Rad). Full-length uncropped original Western blot images are provided in Supplementary File 2.

**Cell viability assays**

5 x 10^4^ cells were plated in FMA medium (described above) in triplicate into 96-well flat-bottom plates and treated with serial dilutions of nutlin-3A (inhibitor of MDM2 to activate TRP53; Cayman Chemical, #18585), etoposide (Ebewe Pharmaceuticals Ltd, A-4866), S63845 (Active Biochem, #6044) ^2^ or ABT-199/Venetoclax (Active Biochem, #A-1231) ^3^. Cell viability was determined at 24 h by staining cells with 2 µg/mL PI (Sigma) and AnnexinV (AnnV) conjugated to Alexa Fluor 647 (WEHI) followed by analysis in a Fortessax20 flow cytometer (Becton Dickinson). Data were analysed using FlowJo^TM^ analysis software. Cell viability was normalised to DMSO vehicle control treated cells.

**RNA sequencing analysis**

All samples were aligned to the mm10 build of the mouse genome using the Rsubread aligner (v 2.0.1) ^4^ with >98% of fragments (read pairs) were mapped to genome. Then all fragments overlapping mouse genes were summarised into counts using Rsubread’s featureCounts function. Genes were identified using Gencode annotation to the mm10 genome (v M25), >83% of mapped fragments were assigned to genes for all samples. Differential gene expression (DGE) analysis was then undertaken using the limma ^5^ (v 3.44.3) and edgeR ^6^ (v 3.30.3) software packages.

Prior to analysis all gender specific genes – Xist and those unique to the Y-chromosome were removed to avoid gender biases and non-protein coding genes, immunoglobulin genes and those genes identified as ‘To be Experimentally Confirmed (TEC)’ were also removed. Expression bases filtering was then performed using edgeR’s filterByExpr function with default parameters. A total of 15,846 genes remained for downstream analysis. Compositional differences between the samples were then normalised using the trimmed mean of M-values (TMM) method ^7^ then transformed to log_2_ counts per million (CPM). The correlation between samples from mice transplanted with HSPCs from each foetal liver was calculated using limma’s duplicate correlation function ^8^. Differential gene expression between the KO and control sample groups was then assessed using linear models which incorporated the aforementioned correlation and robust empirical Bayes moderated t-statistics with a trended prior variance (robust limma-trend pipeline with duplicate correlation ^9^. DGE was assessed relative to a fold-change threshold of 1.2 using limma’s treat function ^10^. The Benjamini and Hochberg method was used to control the false discovery rate (FDR) below 5%. A total of 3,218 genes were found to be significantly differentially expressed between the two samples.

Pathway analyses of the Gene Ontology (GO) and Kyoto Encyclopedia of Genes and Genomes (KEGG) were conducted using limma’s goana and kegga functions, respectively. Analysis of the Molecular Signatures Database Hallmark gene sets was achieved using limma’s fry gene set test. The mean-difference plot was generated using limma’s plotMD function. All heatmaps were created using the pheatmap software package.

**ChIP sequencing analysis**

The previously published ChIP-seq data are available from Gene Expression Omnibus, accession number GSE133514 ^11^. All FastQ files were downloaded and aligned to the mm10 build of the mouse genome using the Rsubread aligner (v 2.10.5) ^4^. At least 90% of reads were successfully mapped for all samples. Following alignment PCR duplicate reads were marked using Sambamba (v 0.6.6).

To generate the coverage plots, the reads aligning to each gene from both the ChIP-seq and RNA-seq data sets were first read into R and the coverage calculated for each sample using the GenomicAlignments package (v 1.31.1). Duplicate reads were excluded for the ChIP-seq samples. The coverage for each sample was then divided by its respective library size multiplied by 1 million to generate counts per million (CPM). The mean was then taken of the replicate samples to give the average coverage for each experimental group. The coverage plots were then produced using the Gviz package (v 1.40.1).

**Supplementary references**

48. Janic A, Valente LJ, Wakefield MJ, Di Stefano L, Milla L, Wilcox S*, et al.* DNA repair processes are critical mediators of p53-dependent tumor suppression. *Nat Med* 2018, **24**(7)**:** 947-953.

49. Kotschy A, Szlavik Z, Murray J, Davidson J, Maragno AL, Le Toumelin-Braizat G*, et al.* The MCL1 inhibitor S63845 is tolerable and effective in diverse cancer models. *Nature* 2016, **538**(7626)**:** 477-482.

50. Roberts AW, Davids MS, Pagel JM, Kahl BS, Puvvada SD, Gerecitano JF*, et al.* Targeting BCL2 with Venetoclax in Relapsed Chronic Lymphocytic Leukemia. *N Engl J Med* 2016, **374**(4)**:** 311-322.

51. Liao Y, Smyth GK, Shi W. The R package Rsubread is easier, faster, cheaper and better for alignment and quantification of RNA sequencing reads. *Nucleic Acids Res* 2019, **47**(8)**:** e47.

52. Ritchie ME, Phipson B, Wu D, Hu Y, Law CW, Shi W*, et al.* limma powers differential expression analyses for RNA-sequencing and microarray studies. *Nucleic Acids Res* 2015, **43**(7)**:** e47.

53. Robinson MD, McCarthy DJ, Smyth GK. edgeR: a Bioconductor package for differential expression analysis of digital gene expression data. *Bioinformatics* 2009, **26**(1)**:** 139-140.

54. Robinson MD, Oshlack A. A scaling normalization method for differential expression analysis of RNA-seq data. *Genome Biology* 2010, **11**(3)**:** R25.

55. Smyth GK, Michaud J, Scott HS. Use of within-array replicate spots for assessing differential expression in microarray experiments. *Bioinformatics* 2005, **21**(9)**:** 2067-2075.

56.. Phipson B, Lee S, Majewski IJ, Alexander WS, Smyth GK. ROBUST HYPERPARAMETER ESTIMATION PROTECTS AGAINST HYPERVARIABLE GENES AND IMPROVES POWER TO DETECT DIFFERENTIAL EXPRESSION. *Ann Appl Stat* 2016, **10**(2)**:** 946-963.

57. McCarthy DJ, Smyth GK. Testing significance relative to a fold-change threshold is a TREAT. *Bioinformatics* 2009, **25**(6)**:** 765-771.

58. Tonc E, Takeuchi Y, Chou C, Xia Y, Holmgren M, Fujii C*, et al.* Unexpected suppression of tumorigenesis by c-MYC via TFAP4-dependent restriction of stemness in B lymphocytes. *Blood* 2021, **138**(24)**:** 2526-2538.
