# Supplementary figures and images for: Deletion of the transcriptional regulator TFAP4 accelerates c-MYC-driven lymphomagenesis
Source: Cell Death Differ. 2023 Mar 9;30(6):1447–56. doi: 10.1038/s41418-023-01145-w (PMC10244435; doi:10.1038/s41418-023-01145-w)

Relating to Figure 2a

Antibody: p53

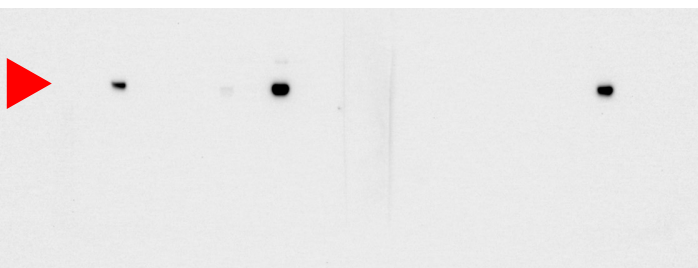

Antibody: p19ARF

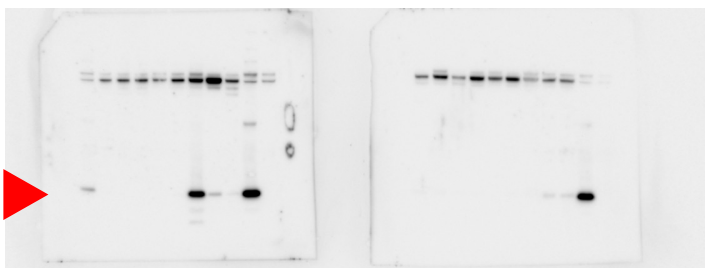

Antibody: HSP70

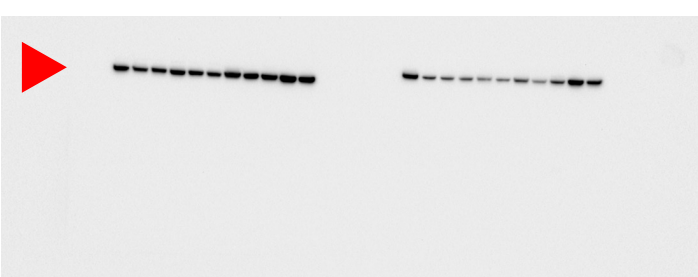

Antibody: TFAP4

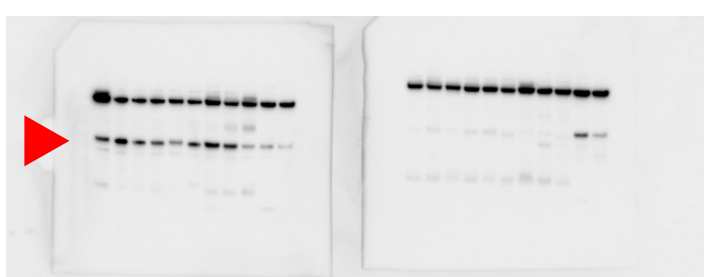

Ladder colorimetric

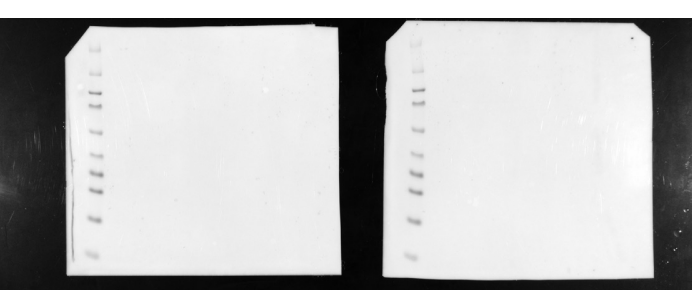

Supplement: Supplementary file 4 — Supplementary File [file 41418_2023_1145_MOESM4_ESM.pdf]
